# Use of the Brazilian version of the Interpersonal Negotiation Strategies Interview (INSI) in a child and adolescent sample: a pilot study

**DOI:** 10.47626/2237-6089-2020-0136

**Published:** 2021-03-31

**Authors:** Tiago Figueiredo, Dídia Fortes, Vanessa Ayrão, Camila Bernardes, Natália Oliveira, Rejane Soares, Raquel Quimas Molina da Costa, Felipe Sudo, Gail Tripp, Maria Antonia Serra-Pinheiro, Paulo Mattos

**Affiliations:** 1 Instituto D’Or de Ensino e Pesquisa Rio de Janeiro RJ Brazil Instituto D’Or de Ensino e Pesquisa (IDOR), Rio de Janeiro, RJ, Brazil.; 2 Instituto de Psiquiatria Universidade Federal do Rio de Janeiro Rio de Janeiro RJ Brazil Instituto de Psiquiatria, Universidade Federal do Rio de Janeiro (UFRJ), Rio de Janeiro, RJ, Brazil.; 3 Instituto do Cérebro Rio de Janeiro RJ Brazil Instituto do Cérebro, Rio de Janeiro, RJ, Brazil.; 4 Okinawa Institute of Science Technology Graduate University Okinawa Japan Okinawa Institute of Science and Technology Graduate University, Okinawa, Japan.

**Keywords:** Social skills, psychometrics, pilot projects, social issues

## Abstract

**Introduction:**

Interpersonal negotiation skills (INS) comprise actions used to solve social situations between interacting individuals involving different needs or desires. These abilities are part of one’s social competence and may be impaired in some psychiatric conditions. There are few validated psychometric tools for measuring INS in the literature. This pilot study aimed to investigate some basic psychometric properties of the Brazilian version of the Interpersonal Negotiation Strategies Interview (INSI) in children and adolescents.

**Methods:**

We developed a new version of the INSI adapted to the Brazilian culture using eight different dilemmas in dyadic situations (with peers and adults), presented visually as drawings on cards. A group of psychologists and psychiatrists chose and adapted the dilemmas formerly proposed by the original version. The same scoring criteria as for the original instrument were used. A total of 20 children and adolescents were included in this pilot study. We investigated test reliability using measures of interrater reliability, test-retest, and internal consistency. The content validity of the INSI was also evaluated by comparison with scores from the Child Behavior Checklist-Revised (CBCL).

**Results:**

Internal consistency and test-retest evaluations were acceptable (rater 1: α = 0.77; rater 2: α = 0.72); the reliability of the instrument was excellent (K = 0.078; intraclass correlation coefficient = 0.98; 95% confidence interval 0.97-0.99); and content validity was strongly significant (p < 0.001).

**Conclusions:**

Preliminary results suggest that this version of the INSI has good interrater reliability and internal consistency and constitutes a promising tool to assess social competence.

## Introduction

Social functioning refers to a comprehensive concept that includes social competence in some domains of life as a defining variable. Social competence comprises the individual’s repertoire of communication skills and interpersonal negotiation skills (INS). INS enable an individual to develop and maintain good interpersonal relationships, express prosocial behavioral patterns, and achieve good acceptance by peers and people from other age groups.^1-3^ INS comprise actions used to solve conflicts evoked when one’s needs or desires disagree with those of others.^4^ This behavior is preceded by cognitive operations comprising perception, interpretation, and response elaboration, considering one’s intention and possible outcomes from interactions with partners.^5^

Use of deficient strategies to negotiate interpersonal conflicts is a critical feature of problematic relationships and strongly contributes to social impairment.^6^ The ability to negotiate successful solutions to social conflicts is a cognitive skill that develops throughout pre-adolescence and early adolescence, hypothesized as a precursor to moral development and prosocial behaviors.^7^ This ability is positively related to empathic understanding, helping behaviors, social competence, and social status.^8-11^

Using observational and interview procedures, Selman et al. proposed the interpersonal negotiation strategies (INS) model of how children and adolescents articulate and use strategies to negotiate interpersonal conflicts.^12,13^ This model focuses on how, in a dyadic context for interpersonal negotiation, one individual deals with another to resolve the disequilibrium that arises between them in particular social conflict situations. The model takes into consideration that context influences negotiation. An adolescent may use a different overall level of interpersonal negotiation with a peer than he/she would use with an adult. It also includes some situations in which the protagonist is attempting a change (initiation), and other situations in which the protagonist is reacting to a change initiated by the other person (reaction). Situations may also vary in terms of whether the other individual has an intimate relationship with the protagonist or not.

The INS model proposes that solving a social dilemma comprises four main stages: 1) comprehension/identification of the problem; 2) feelings triggered by the social problem-solving process 3) choosing the strategy to be used; and 4) justification (prospecting outcomes). The pattern of responses presented in each of these steps is the variable to be assessed. This pattern is classified according to the level of complexity of the ability to coordinate conflicting individual perspectives. The original model considers four levels of complexity: impulsive, unilateral, reciprocal, and collaborative. It is expected that the level of complexity will progressively evolve throughout the stages of development, already reaching a satisfactory level during adolescence.^11^ Based on the INS theoretical model, the Interpersonal Negotiation Strategies Interview (INSI) is a psychometric instrument developed by Selman et al.^1^ It is a structured dilemma-discussion interview involving hypothetical social conflict situations used to assess the four steps of the INS process (Table 1).^1^ In the original instrument, dilemmas are presented verbally to the examinee.

**Table 1:** Four steps according to the INS model1

Step	Description
Step 1: Definition and comprehension of problem	This step evaluates the ability to define with accuracy the nature of the social problem. This step changes from a definition that focuses on physical and concrete aspects of the problem (e.g., “He is jealous of the new friend”) to one concerned more with interpersonal relationships (e.g., “They have a problem and need to decide whether to take a new friend with them”). The manner in which the problem is defined helps clarify the goal of the negotiation process.
Step 2: Identifying the emotions generated by the dilemma exposed	This step evaluates the ability to identify and name the emotions generated by the conflict situation. This is an important step to be assessed because it enables evaluation of the primitiveness (e.g., “he is sad”) or complexity (e.g., “he is distressed”) of emotion processing and helps to assess the participant’s assertive skills.
Step 3: Generating strategies and selecting a specific strategy	This step refers to the ability to think of more than one potential strategy that may solve the problem presented. It can be useful in assessing flexibility (coordinating reciprocal social perspectives) or insistence on the same pattern (simple physical strategies), and also to assess the ability to choose a particular strategy. This capacity depends on the ability to anticipate the consequences of the alternatives and to plan further alternatives that may be implemented, if necessary, to accomplish the goal. It is possible to capture some patterns of plans that change from little ability to plan longer consequences (avoidant, impulsive) to more complex strategies that consider longer outcomes (attending both perspectives).
Step 4: Justification of strategy and evaluating the outcomes	This final step refers to the ability to evaluate the outcomes of specific negotiation strategies and processes based on verbal justification of the strategy selected. This step is important to assess the conscious choice process and to evaluate whether the subject considered any perspective to maintain the relationship.

INS = interpersonal negotiation strategies.

Since its creation and validation, the INSI has been used as a psychometric tool to assess INS in different samples. Social dilemmas have sometimes been modified according to the goals of the study. Some authors have used pictures and even videos to portray dyadic social situations.^14-16^ However, scoring criteria have always followed the original proposal for assessment of four steps according to the original INS model.^17^

Children and adolescents who have or are at risk of psychiatric disorders often experience difficulties in social competences; these difficulties should be investigated by mental health professionals because of their potential adverse outcomes. Social competence in children and adolescents is widely assessed by collateral informants, mainly parents and teachers; while admittedly important, this strategy can, however, be heavily influenced by a myriad of biases.^18-20^ Also, interventions for impairments in social functioning generally involve strategies based on the assumption that the person did not acquire the social skills along their developmental stages.^21^ However, collateral report may not be informative for subgroups of children with social deficits that are mediated by specific conditions such as attention deficit/hyperactivity disorder (ADHD). Some childhood psychiatric conditions are associated with performance deficits rather than with skills acquisition deficits.^22,23^

Considering the relevance and scarcity of standard psychometric tools that assess social competence domains (social skills knowledge and performance), we consider the INSI a promising assessment tool for both research and practice. We believe that the Brazilian version of the INSI can open new perspectives for evaluation of these abilities in this particular culture. In this pilot study, we report the psychometric properties (internal consistency, reliability, and validity) of the Brazilian version of the INSI in a sample of patients with varied conditions from a children’s psychiatric center.

## Methods

### Participants

A total of 20 participants were examined (children = 13, age range 8-12 years-old; adolescents = 7, age range 13-17 years-old). All of them were recruited through the outpatient unit for neuropsychological assessment at the Instituto D’Or de Ensino e Pesquisa (IDOR), in Rio de Janeiro, Brazil. All participants had been referred because of learning and/or behavioral problems and underwent a sequential order of evaluation that comprised psychiatric assessment, INSI interview, and neuropsychological and language assessments. Participants had middle-high socioeconomic status according to the occupation and education level of their parents (Hollingshead AB, Four-factor index of social status, unpublished manuscript, New Haven, Yale University, 1975). This study was approved by the IDOR ethics committee. All participants and their respective parents provided written informed consent.

Exclusion criteria were: a) cognitive impairment defined as an intelligence quotient (IQ) < 80; b) presence of autism spectrum disorders (ASD) and/or autistic traits; c) communication disorders (language development disorder); d) psychosis or acute mania; or e) genetic syndromes or neurological conditions (e.g., epilepsy). All interviews were conducted by a board-certified psychiatrist using criteria from the Diagnostic and Statistical Manual of Mental Disorders, 5th edition (DSM-5).^24^

### Measures

#### Child and adolescent psychiatric evaluation

A semi-structured clinical interview was conducted with the participants’ parents using the adapted version of the Kiddie Schedule for Affective Disorders and Schizophrenia for School-Aged Children (K-SADS). The K-SADS (2017 update) was used to assess the nature, onset, course, duration, severity, and impairment of current and past psychopathology episodes in the children and adolescents included in the sample, according to DSM-5 criteria.^24^ The Brazilian version of the K-SADS has shown good convergent validity with the original version.^25^

Parents provided a collateral report regarding anxiety and ADHD symptoms through the parent’s version of Screen for Child Anxiety Related Emotional Disorders (SCARED) and the Swanson, Nolan, and Pelham-IV Questionnaire (SNAP-IV), respectively.^26,27^ The Portuguese version of the STAI has shown high internal consistency, with Cronbach’s α = 0.89. The SNAP-IV was developed to assess symptoms of ADHD and oppositional defiant disorder (ODD) according to DSM-IV diagnostic criteria.

All participants completed the Screen for Child Anxiety Related Emotional Disorders (SCARED) and the Children’s Depression Inventory (CDI) to investigate the presence of anxiety and depressive symptoms, respectively.^28,29^ Similarly to the original version, the Portuguese version of SCARED comprises 69 items that aim to assess different dimensions of anxiety-related problems in children: separation anxiety, generalized anxiety disorder, panic disorder, social phobia, specific phobia, obsessive-compulsive disorder, and post-traumatic stress. The CDI is internationally considered as the most widely used instrument to assess depressive symptoms in children and adolescents, in both clinical and research contexts.

#### Social problem evaluation

Parents filled out the Child Behavior Checklist-Revised (CBCL) in order to record social problems.^30^ Based on previous exploratory analyses, using the items from the CBCL Social Problem Scale, it was possible to assess two domains of social functioning “Social Immaturity” and “Peer Rejection.”^31^ We opted to use correlations between four items of the “Peer Rejection” subscale (25, 37, 38, and 48) and INSI scores in further analyses, considering the close relationship between Interpersonal Negotiation ability and outcomes related to Peer Acceptance in social functioning assessment.^32^

#### Cognitive assessment and language evaluation

In order to exclude primary comprehension bias, such as cognitive and/or communication deficits, the Weschler Abbreviated Scale of Intelligence (WISC-IV) was administered to participants for IQ measurement. Also, the Brazilian versions of the Protocole Montréal d`Évaluation de la Communication and the Faux Pas and Teste de Coerência e Inferência Local were administered to all participants to assess language development and social cognition features.^33-35^

#### The Brazilian version of the INSI and scoring: identification of domains and construction of dilemmas

Four psychiatrists and two psychologists with extensive experience in children’s and adolescents’ mental health were invited to participate in two separate meetings to develop the social dilemmas to be used in the structured interview. At the first meeting, four dilemmas for evaluation of children (8-12 years old) and four for evaluation of adolescents (13-17 years old) were chosen from the twelve dilemmas from the original version of the INSI (covering seven situations involving adolescents and five involving children).^1^ Criteria for selection of dilemmas included the need to choose two dilemmas portraying social situations involving peers and two dilemmas involving cases with adults. For each of the relationship domains, one situation should represent an intimate relationship. The professionals’ choice of dilemmas was based on their individual judgment of which dilemmas were most congruent with the Brazilian social culture (for example, dilemmas that refer to work situations for adolescents were excluded, since middle-income adolescents do not work in Brazil). There were no ties in dilemma selection, although it had been agreed in advance that, in the event of a tie, the first author would vote for a tiebreaker.

Once the dilemmas had been selected, they were adapted to the Brazilian culture. The texts for the hypothetical dilemmas were not written by translation from the English, but utilized the primary nuclei of each history (a dyadic social problem). The final versions of each of the dilemmas used in the interview are presented in the Appendix and available as online-only supplementary material.

At the second meeting, the scoring criteria to debrief the four steps of evaluation were discussed together with their corresponding scoring criteria adapted from the original INS model. Following the original model, responses should be scored on a 4-point scale (0-3) for each step. The scoring criteria are shown in Table 2. The sum of the total score represents the global INS index (INSI total score).

**Table 2:** Scale of response scores according to categories adapted from Selman et al.1

Scoring category	Numeric score value
Definition and comprehension of problem	0 – No reference to the problem; Comprehends the problem with wrong assignments; just repeats the interviewer’s narration; 1 – Understands the problem from a one-way perspective; one perspective is neglected; 2 – Considers both perspectives, but focuses on just one perspective (one of the two persons has priority); 3 – Understands the dilemma as a shared problem and considers both perspectives/needs/desires;
Identifying the emotions generated by the dilemma exposed	0 – Does not refer to any emotions or refers to incongruent feelings; 1 – Simple and unidimensional feelings expressed in a self-protective way; 2 – Simple and one-dimensional feelings expressed in an empathic way; 3 – Complex or multiple feelings that consider the other’s needs/perspective.
Generating strategies and selecting a specific strategy	0 – Physical, noncommunicative methods 1 – One-way strategies or requests; one-way commands and assertions; 2 – Reciprocal communication including trades, exchanges, verbal persuasion, suggestions that convince and protect subjective interests; 3 – Strategies that focus on collaboration, need for integration of the interests.
Justification of strategy and evaluating the outcomes	0 – No justification or anticipation of consequences are expressed/considered; 1 – Self-protective justification; 2 – Empathic concerns without considering long-term consequences to the relationship; 3 – Expression of concerns for long-term effects on the relationship.

**Figure 1:**
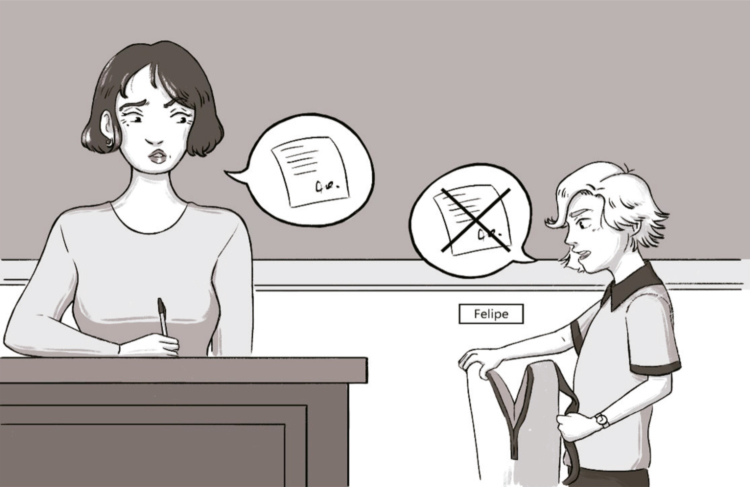
Example of a card portraying one of the dilemmas for children. Dilemma: “Felipe/Maria Clara is attending a class with a substitute teacher. He/she needs to leave earlier today because he/she has an important appointment. But he/she realizes that he/she has forgotten the authorization signed by his/her mother. When he/she approaches his/her teacher, she says Felipe/Maria Clara must have the document in order to leave, because this is the school’s rule.”

The INSI application included reading the short history of each dilemma and asking semi-structured questions regarding problem-solving steps. The dilemmas were presented in the same order for all participants. The protagonist in each dilemma always had the same gender as the participant (there were always two options for each dilemma). We used cards portraying the dilemmas to mitigate attentional or working memory deficits (example in Figure 1). Each scene was described aloud by the examiner while presenting the card to the examinee. Answers were audio-recorded and transcribed. The total score of the Brazilian version of INSI was used for subsequent analysis.

## Data collection and analysis

The participants were enrolled on a non-randomized sequential order of evaluation. The total evaluation time lasted three days, with appointments lasting between 2 and 4 hours. The first day included the psychiatric assessment, collection of participant’s and parents’ reports (using the questionnaires), and the INSI interview. On the second day, the participant underwent the neuropsychological tests. On the last day, the participant underwent the language assessment.

Two previously trained interviewers participated in collecting the INSI data. Two blinded raters (designated raters 1 and 2) scored the INSI responses for the four steps, each providing total scores for the same subjects independently. Raters had separate meetings with two authors (TF and PM) in order to clarify items and check for consistency using real examples from the sample. The total score of the Brazilian version of the INSI was used for subsequent analysis.

The normality of the data distribution was assessed using the Kolmogorov-Smirnov test. The internal consistency was estimated using Cronbach’s α and values larger than 0.70 were considered acceptable.^36^ Intraclass correlation coefficients (ICC [2,1]) and the Kappa (K) concordance test were applied to measure interrater and test-retest reliability, respectively.^37^ These results were interpreted as follows: < 0.5 = poor reliability, between 0.5 and 0.75 = moderate reliability, between 0.75 and 0.9 = good reliability, and > 0.90 = excellent reliability.^37^ The significance level was set at α=0.05. In addition, measurement error was evaluated using standard error of measurement (SEM), as follows: SEM = SD x Ö(1-ICC); where: SD = Standard deviation.^38^ The results for Internal Consistency, ICC, and test-retest were used to evaluate the reliability of the Brazilian Version of the INSI.

The scores of CBCL items that assess social problems were used to test the content validity of the INSI. Construct validity was investigated by examining the relationship between the total INSI score and the CBCL score. Spearman’s correlation coefficients were used to investigate correlations between INSI and CBCL scores. Due to a need to calculate the number of participants for an additional analysis involving ADHD-related social problems, we separated participants into two groups: the ADHD group and the Clinical Control group. Next, we conducted a comparison between mean INSI performance (total scores) of both groups (ADHD and clinical controls) using Pearson’s *t *test. The IBM Statistical Package for Social Sciences version 25 (SPSS v. 5), Stata 16 software (Stata Corporation), and MS Excel 365 were used for all these analyses.

## Results

### Sample characteristics

The total sample included twenty children and adolescents. Thirteen (65%) were male. The mean age and IQ of participants was 11.76 (SD = 2.86) years and 104.38 (SD = 11.43), respectively. None of the participants presented language impairment or abnormal performance in Theory of Mind (ToM) tests. Thirteen subjects fulfilled ADHD diagnostic criteria. One of these had comorbid ODD symptoms and nine presented high levels of anxiety. Six of the remaining seven participants had learning disorders and one had a mood disorder. The sociodemographic characteristics and prevalence of psychiatric problems are shown in Table 3.

**Table 3:** Sample characteristics

Variable	Result (n = 20)
Sex	
Male	13 (65%)
Female	11 (35%)
Age range	8-17
IQ scores	104.7 (11.33)
Psychiatric problems	
ADHD	13
ODD	1
Anxiety symptoms	13
Depressive symptoms	6
Learning disorders	8

ADHD = Attention-deficit Hyperactivity Disorder; ODD = Oppositional Defiant Disorder.

### Internal consistency and interrater reliability

For both raters, internal consistency was acceptable (rater 1: α = 0.77; rater 2: α = 0.72). Results for internal consistency are shown in Table 4. The Kappa (K) agreement coefficient for total INSI score was 0.786 (p < 0.001). The overall reliability of the instrument was substantial (ICC = 0.98; 95% confidence interval [95%CI] 0.97, 0.99). Most of the subitems presented moderate reliability, but some items yielded low ICC values (Comprehension of dilemma item 2: ICC = 0.40; 95%CI -0.48, 0.75; Strategy of situation item 2: ICC = 0.30; 95%CI -0.68, 0.71; Comprehension of situation item 3: ICC = 0.19; 95%CI -1.00, 0.67; and Strategy of situation item 4: ICC = 0.28; 95%CI -0.74, 0.71). On average, reliability indices for comprehension (ICC = 0.63; 95%CI 0.09, 0.85) and emotions (ICC = 0.58; 95%CI -0.02, 0.83) items were lower than for the other INSI subtests. Table 4 shows the results for each dilemma.

**Table 4:** Results of test-retest analyses

Items/rater	Mean (SD)	ICC	95%CI	SEM
Dilemma 1				
Comprehension				
R1	1.9 (0.94)	0.71	0.28-0.88	0.74
R2	1.95 (0.86)			
Strategy				
R1	1.66 (0.91)	0.54	-0.04-0.81	0.91
R2	2.04 (0.86)			
Justification				
R1	1.19 (0.98)	0.69	0.22-0.87	0.82
R2	1.23 (0.99)			
Emotions				
R1	1.42 (0.74)	0.51	-0.15-0.80	0.87
R2	1.71 (1.00)			
Dilemma 2				
Comprehension				
R1	1.71 (0.78)	0.40	-0.48-0.75	0.92
R2	1.51 (0.81)			
Strategy				
R1	1.80 (0.68)	0.30	-0.68-0.71	0.93
R2	2.04 (0.86)			
Justification				
R1	1.66 (0.79)	0.72	0.30-0.88	0.67
R2	1.66 (0.96)			
Emotions				
R1	1.76 (0.76)	0.68	0.23-0.87	0.69
R2	1.52 (0.92)			
Dilemma 3				
Comprehension				
R1	1.47 (0.87)	0.19	-1.00-0.67	1.04
R2	1.28 (0.56)			
Strategy				
R1	1.85 (0.91)	0.70	0.24-0.89	0.77
R2	1.80 (0.98)			
Justification				
R1	1.33 (0.65)	0.67	0.19-0.87	0.68
R2	1.42 (1.07)			
Emotions				
R1	1.66 (0.73)	0.72	0.32-0.88	0.62
R2	1.47 (0.87)			
Dilemma 4				
Comprehension				
R1	1.38 (0.67)	0.61	0.04-0.84	0.64
R2	1.23 (0.70)			
Strategy				
R1	1.95 (0.97)	0.28	-0.74-0.71	1.24
R2	2.23 (0.99)			
Justification				
R1	1.74 (1.03)	0.64	0.09-0.85	0.94
R2	1.38 (1.07)			
Emotions				
R1	1.76 (0.62)	0.69	0.26-0.87	0.53
R2	1.28 (0.65)			
Comprehension (total score)				
R1	6.42 (2.13)	0.63	0.09-0.85	1.86
R2	6.00 (1.87)			
Strategy (total score)				
R1	7.28 (1.95)	0.79	0.15-0.93	1.57
R2	5.71 (2.96)			
Justification (total score)				
R1	5.66 (2.41)	0.76	0.42-0.90	2.05
R2	4.40 (3.54)			
Emotions (total score)				
R1	6.61 (2.45)	0.58	-0.02-0.83	2.73
R2	4.40 (3.54)			
INSI total score				
R1	26.38 (6.27)	0.98	0.97-0.99	1.34
R2	25.85 (6.47)			

95%CI = 95% confidence interval.

The dilemmas applied to children and adolescents were analyzed together because of their similar scoring steps.

### Validity

Construct validity (social problems) was analyzed using the total score for the “Peer Rejection” subscale of the CBCL. This was assessed as the correlation between the INSI total score and the correspondent value for the sum of specific CBCL item scores. The INSI total score was found to be robustly, significantly, and inversely correlated with the “peer rejection” subscale of the CBCL (high INSI scores were associated with fewer social problems in parents’ reports), r = -0.86, p < 0.001.

### INS scores between the two groups and sample size reliability

Regarding INSI performance, the mean total score in the sample analyzed was 25.3 (SD = 6.81). The mean INSI total scores were 22.72 (SD = 6.04) in the ADHD group and 29.5 (SD =5.95) in the clinical controls group. The difference in INSI performance between the two groups was statistically significant [t(17.3) = -2.69, p < 0.05].

The power of the sample size was analyzed separating the subjects into two groups: the ADHD group and the clinical controls group. A *t* test was performed considering the mean INSI performance scores of both groups. The significance was α = 0.10 and the test power was 0.80.

## Discussion

This study aimed to report the psychometric properties of the Brazilian version of the INSI, and preliminary findings obtained in the sample analyzed. This pilot study is part of a larger research project on social competence of children and adolescents and evaluates the performance of an ADHD subset of the sample. Our results suggest that the Brazilian version of the INSI has good interrater reliability, internal consistency, and strongly significant validity with another validated instrument that assesses child and adolescents’ social problems. Preliminary findings indicated deficient performance on INSI in the ADHD group when compared with the comparison group’s performance.

Social competence refers to a broad concept that involves a spectrum of social skills. Among them, the ability to solve interpersonal conflicts involves some social-cognitive skills such as problem recognition (definition), identification of own behavior and others’ intentions and emotions, development of strategies to solve the conflict and managing the immediate and later outcomes. Development of INS during pre-adolescence and adolescence is an essential part of social competence. This process gives individuals an adaptative transition in the way they deal with others, from a position of self-interest to one of collaboration with another for the sake of mutual interest and intimacy.

Children and youth who have or are at risk of some psychiatric disorders experience critical difficulties in developing and maintaining good interpersonal relationships.^19^ This fact underscores the need for continuous improvement in the assessment of social competence difficulties. An important theoretical perspective refers to a distinction between deficits in acquisition of social skills and deficits in performance of social skills.^39^ Consideration of these perspectives enables the nature of specific social skills difficulties to be assessed in order to design specific treatment strategies. A wide number of authors currently use social problem rating scales to evaluate child social competence and measure their social functioning deficits by multi-informant reports (parents and teachers). Although some specific scales address social competence in children, some studies highlight significant discrepancies between multi-informant scores.^40-43^ We recognize the importance of the family and teachers’ perceptions of child social competence for assessment. Still, we believe that these reports make a greater contribution to measuring the impairment level than to revealing the target of individual deficits.

The INSI constitutes a dynamic assessment tool that disentangles the different steps involved in interpersonal negotiation, including different perspectives on hypothetical social situations. It demands that the child selects the social skills required and coordinates the best way to use them, considering possible future outcomes, in different social contexts. We believe that this type of direct evaluation allows complementary assessment of both domains of social abilities (social skills acquisition and performance) and can be useful to create individual approach strategies. Moreover, use of quantitative analyses on four different, although related, levels (definition of the problem, action taken, justification and consequences of the strategy chosen, and complexity of feelings expressed) allows an in-depth insight into the individual’s competence.

The Brazilian version of the INSI can be considered an instrument with adequate psychometric properties for use. The internal consistency was moderate in some specific items. The sample assessed was characterized as having a significant number of subjects with ADHD. We believe that the heterogeneity and the small sample size may have contributed to the moderate internal consistency. Also, the test’s inherent properties are not linear due to the different hypothetical situations presented. The statistically significant difference in INSI scores between the ADHD subgroup and clinical controls reinforced the construct validity of impaired social performance. These preliminary results support the need to expand the sample under analysis in order to obtain new findings that contribute to better understanding of the mechanisms involved in the social competence of children and adolescents, especially those with neurodevelopmental disorders.

We did not include individuals with ASD because their significant impairment in ToM would jeopardize analysis of their negotiation strategies. However, we must investigate the ToM measures as covariates in future studies with larger samples, since ToM is an essential part of social cognition and, therefore, an important part of negotiation abilities.^44,45^ Since this was a pilot study, a small sample was analyzed. The ADHD group’s deficient performance in the INSI constitutes a preliminary finding, and we intend to replicate and deepen this analysis in further studies.

### Limitations and further perspectives

Our study has some limitations inherent to a pilot study. Regarding sample characteristics, we analyzed a convenience sample and our small sample size warrants caution in the interpretation of results, but these are similar to other studies.^45^ Further analysis should include test-retest processes and possible correlations with intelligence level, gender, and age, demanding a much larger sample. Ultimately, we believe that the majority of ADHD subjects in the sample enrolled in this analysis can be considered a confounding factor in the analysis of the test’s convergent validity.
