# Supplementary material for: Use of the Brazilian version of the Interpersonal Negotiation Strategies Interview (INSI) in a child and adolescent sample: a pilot study
Source: Trends Psychiatry Psychother. 2021 Mar 31;44:e20200136. doi: 10.47626/2237-6089-2020-0136 (PMC9972889; doi:10.47626/2237-6089-2020-0136)
Supplement: Supplementary file 1 [file 2238-0019-trends-v44-e20200136-suppl01.pdf]

http://dx.doi.org/10.47626/2237-6089-2020-0136

Appendix - Final version of each dilemma for the interview

	**Children (8-12 years)**	**Adolescents (13-17 years)**
Dilemma 1:	"Bruno/Debora and Samuel/Ana are classmates, but they are not close to each other. The teacher set the class a new activity to be done in pairs, in which students have to write a text about an African theme. Bruno/Debora wants to write about African wildlife, but Samuel/Ana wants to write about African tribes, like the Pigmies".	"João/Júlia has a class every Friday that finishes at 2 pm. He/She is anxious because he/she has an important appointment after school and needs to leave in time. Fifteen minutes before the end, the teacher tells students to prepare a text and tells them that no one can leave before all students finish the activity. João/Júlia presumes that this will delay his/her departure".
Dilemma 2:	"Bernardo/Laura and Caio/Carol are best friends. They're planning for the weekend and they have decided to go to the cinema. Bernardo/Laura wants to invite his/her new friend to go with them, but Caio/Carol doesn't like this idea.	"Daniel/Amanda has a romantic relationship with Paulinha/Lucas, but he/she wants to flirt with other girls/boys. He/she doesn't agree with this".
Dilemma 3:	"João Victor/Mariana have an important football/volleyball competition tomorrow. He/She needs to use the school interval to train with his/her team. The class before the interval is math and the teacher told him/her that his/her math notes are not good and suggested an after class extra activity in order to help him improve. But João Victor/Mariana doesn't want to miss training for the competition".	"Igor/Juliana agreed with his/her mother to go to the mall to buy new clothes for him/her for the weekend party. When they were leaving, his/her mother told him/her she had invited her friend and her son/daughter to go with them. But Igor/Juliana can't stand being around this boy/girl".
Dilemma 4:	"Felipe/Maria Clara is attending a class with a substitute teacher. He/she needs to leave earlier today because he/she has an important appointment. But he/she realizes that he/she has forgotten the authorization signed by his/her mother. When he/she approaches his/her teacher, she says Felipe/Maria Clara must have the document in order to leave, because this is the school's rule."	"Miguel/Maria and Pedro/Livia have been a pair for biology class since the beginning of the year and often use a computer to search themes for their schoolwork. They always take turns: one uses the computer and the other writes down the manuscript. This would be Miguel's/Maria's turn to use the computer, but Pedro/Livia said that his/her wrist is aching and asked Miguel/Maria if he/she could use the computer instead. But Miguel/Maria doesn't want to miss the opportunity to use the computer because he/she likes it much more".
